# Is Gluten the Only Culprit for Non-Celiac Gluten/Wheat Sensitivity?

**DOI:** 10.3390/nu12123785

**Published:** 2020-12-10

**Authors:** Maria Gloria Mumolo, Francesco Rettura, Sara Melissari, Francesco Costa, Angelo Ricchiuti, Linda Ceccarelli, Nicola de Bortoli, Santino Marchi, Massimo Bellini

**Affiliations:** Gastrointestinal Unit, Department of Translational Sciences and New Technologies in Medicine and Surgery, University of Pisa, 56124 Pisa, Italy; g.mumolo@int.med.unipi.it (M.G.M.); sara.melissari@gmail.com (S.M.); francesco.costa@int.med.unipi.it (F.C.); a.ricchiuti@int.med.unipi.it (A.R.); l.ceccarelli@ao-pisa.toscana.it (L.C.); nicola.debortoli@unipi.it (N.d.B.); s.marchi@med.unipi.it (S.M.); mbellini@med.unipi.it (M.B.)

**Keywords:** gluten, gluten-free diet, low FODMAP diet, celiac disease, non-celiac gluten/wheat sensitivity, irritable bowel syndrome

## Abstract

The gluten-free diet (GFD) has gained increasing popularity in recent years, supported by marketing campaigns, media messages and social networks. Nevertheless, real knowledge of gluten and GF-related implications for health is still poor among the general population. The GFD has also been suggested for non-celiac gluten/wheat sensitivity (NCG/WS), a clinical entity characterized by intestinal and extraintestinal symptoms induced by gluten ingestion in the absence of celiac disease (CD) or wheat allergy (WA). NCG/WS should be regarded as an “umbrella term” including a variety of different conditions where gluten is likely not the only factor responsible for triggering symptoms. Other compounds aside from gluten may be involved in the pathogenesis of NCG/WS. These include fructans, which are part of fermentable oligosaccharides, disaccharides, monosaccharides and polyols (FODMAPs), amylase trypsin inhibitors (ATIs), wheat germ agglutinin (WGA) and glyphosate. The GFD might be an appropriate dietary approach for patients with self-reported gluten/wheat-dependent symptoms. A low-FODMAP diet (LFD) should be the first dietary option for patients referring symptoms more related to FODMAPs than gluten/wheat and the second-line treatment for those with self-reported gluten/wheat-related symptoms not responding to the GFD. A personalized approach, regular follow-up and the help of a skilled dietician are mandatory.

## 1. Introduction

**Figure 1:**
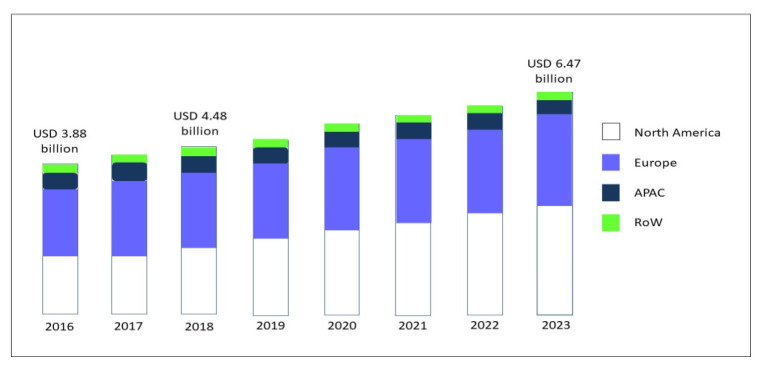
GF product market, by region, during the forecast period of 2016 to 2023, modified from Research and Markets, Report May 2019 [2]. Abbreviations: GF: gluten-free; APAC: Asia-Pacific countries; RoW: rest of the world.

Over the last 30 years, the gluten-free diet (GFD) has gained increasing popularity associated with an exponential growth in the sales of gluten-free (GF) products [1]. The global market for GF food, driven by North America and Europe, but now spreading across the Asia-Pacific countries (APAC), was valued at USD 3.88 bn in 2016, and is foreseen to expand to USD 6.47 bn in 2023, at a compound annual growth rate (CAGR) of 7.60% [2] (Figure 1).

In the USA, a follow-up analysis of the National Health and Nutrition Examination Survey (NHANES) revealed that self-adoption of a GF diet without a diagnosis of celiac disease (CD) tripled from 2009–2010 (prevalence 0.52%) to 2013–2014 (prevalence 1.69%) [3] and NPD’s Dieting Monitor, which tracks nutrition-related issues of consumers, in 2013 reported that nearly 30% percent of adults claimed to cut down on or avoid gluten [4]. In Italy, where bread and pasta are the foundation of food culture, is in the vanguard of the European GF sector with the range of products jumping from 280 in 2001 to the current 6500 and a market amounting to EUR 320 million, of which only 215 are dispensed on prescription for celiac patients. The launch of innovative products containing no or less gluten and dominated by the bakery product segment is on the rise and contributes to boosting this industry. Suppliers continue to invest in innovation to improve taste, texture and overall quality in GF formulations.

The GFD is recommended as lifelong treatment for CD. However, neither government awareness campaigns and initiatives nor the improvement of diagnostic tools and increasing prevalence of CD [5] account for the overwhelming adoption of a GF lifestyle. Clinical application of GFDs continues to escalate as a therapeutic option for non-celiacs who seem to react negatively to gluten ingestion, are trying to lose weight [6] or simply want to reduce bloating after meals [7]. The reasons given for a self-imposed GFD include irritable bowel syndrome (IBS) and lactose intolerance [8]. Other persons spontaneously limit or eliminate gluten intake as a “healthy” dietary regimen without previous clinical tests due to the widespread consumer interest in free-from products and the growing adoption of specific eating patterns in pursuit of health and wellness [9]. In an Australian cross sectional population survey [10], symptomatic wheat avoidance was highly correlated with dairy avoidance, female gender and lesser and greater receptiveness to conventional and complementary medicine, respectively. While perception of the potential harm and expected benefits of gluten consumption/avoidance are high, real knowledge of gluten and GF-related implications for health is scarce. An American survey [11] found that over 30% of respondents had no specific reason for adopting a GF regimen, while less than 10% self-reported gluten sensitivity (GS). The other reasons were a healthy lifestyle, improvement of intestinal health or the presence of a gluten-sensitive family member. Different factors lie at the basis of the GF movement, mostly driven by non-scientific sources of information. While Google searches containing “low carb” and “low fat” have declined since 2004, worldwide searches for “gluten” showed a sharp upward trend, reaching the peak of food concerns from 2005 to 2014. From then on, they have remained generally steady. In Italy, an increase in the number of searches was observed until mid-2019, then there was a decline. This far exceeds lactose, genetically modified organisms (GMO) and palm oil, with ratios approaching 16:1, 6:1 and 2:1, respectively. Marketing campaigns aimed at extending the appeal of GF to every health-conscious consumer despite the high costs of products. Moreover, athletes and celebrities, together with mass media messages and social network platforms, all contribute to increasing awareness of gluten intolerance and fuel the interest in dietary treatments. Consumers commonly select GF products from aisles in major supermarkets and health food shops [12]; for many consumers, the front of package claims are more important determinants of GF product choices [13] than nutritional labeling [14]. Several studies have shown an excessive intake of fats and carbohydrates, with a lower consumption of dietary fibers, in CD patients on a GFD [15]. Moreover, clinically relevant deficiencies of iron, vitamin D, vitamin B6 and zinc have been reported in CD patients during treatment with a GFD, whereas data on deficiencies of vitamin B12, folic acid, calcium and magnesium are controversial [16]. The alarming discrepancy between media claims and scientific evidence [17] drives the motivation and reinforcement of people’s commitment to avoiding gluten, generating a great deal of confusion and misconceptions [18]. This article is aimed at discussing the actual role of gluten in non-celiac gluten/wheat sensitivity (NCG/WS).

## 2. What about Gluten?

### 2.1. Gluten Structure and Genetics

**Figure 2:**
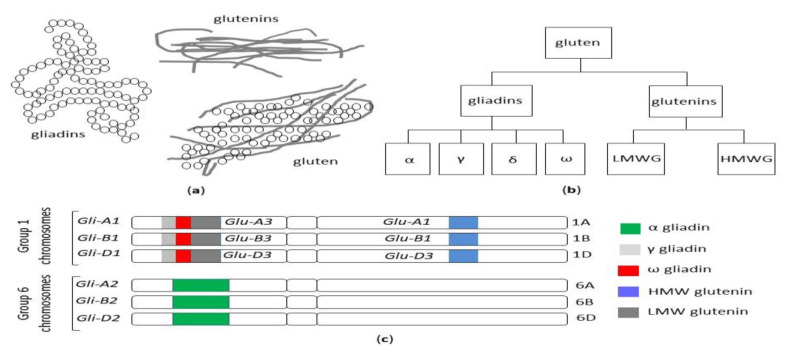
(**a**) The structure of the gluten protein gluten network. (**b**) The classification of wheat gluten proteins. (**c**) Gliadin and glutenin loci in *Triticum aestivum* (AABBDD), modified from Sharma, 2020 [26]. HMW: high molecular weight, LMW: low molecular weight.

The term gluten collectively refers to a family of storage proteins, formally known as prolamins, naturally occurring in wheat, barley and rye, and their cross-bred grains [19]. Although oats also contain prolamins (avenins), they represent a small (10 to 15%) proportion of total protein content, in comparison with 80 to 85% of wheat gliadins. Moreover, avenins contain less proline than the other prolamins, are more easily digested and the peptides show less affinity for MHC II peptides encoded by HLA DQ2.5 haplotypes [20]. These properties could make oats generally safe for celiac patients, although individual hypersensitivity in some celiac patients can occur [21,22], and some varieties may display immunogenicity/toxicity [23,24]. The wheat prolamins are termed gliadins, monomeric proteins divided into four distinct subtypes, referred to as alpha, gamma, delta and omega gliadins and glutenins, and polymeric proteins composed of high (HMW-GS) and low molecular weight subunits (LMW-GS) linked by disulfide bonds [25] (Figure 2a,b).

A significant proportion of prolamins are represented by repetitive sequences of glutamine and proline. The various wheat varieties differ in terms of prolamin molecular weight and microstructure (junction density, branching rate, lacunarity). These characteristics influence the strength of the network and the dough quality and, in turn, determine the tensile and cooking properties [19,25,27]. Due to its unique biochemical and functional features (water-binding and visco-elastic properties, gas retention), gluten is essential for baking but also widely used as an additive in processed food.

Besides its commercial value, the detrimental effects of gluten on human health have been described, mediated by immunological or toxic reactions [28]. Due to the high number of glutamine- and proline-rich periodic sequences, gluten peptides are highly resistant to gastric and intestinal proteolytic degradation, thus giving rise to potentially immunogenic fragments. In addition, gluten alters intestinal permeability, promotes oxidative stress, exerts cytotoxic and pro-inflammatory effects and negatively affects the microbiome; cell apoptosis is increased and cell differentiation is reduced [29]. In celiac patients carrying HLA DQ2/DQ8 haplotypes, gluten triggers an innate, as well as adaptative, Th1-driven immune response, amplified by transglutaminase-mediated synthesis of negatively charged glutamate residues from glutamine [26]. Since the Neolithic Age agricultural revolution, 10,000 years ago, ancient grasses have been domesticated and spread from the Fertile Crescent of the Middle East westward through Europe [30]. Agricultural techniques increased the abundance and availability of wheat, but it is only in the past 500 years that the gluten content of foods containing wheat has significantly increased. Modern hexaploid wheat cultivars have three different genomes (A, B and D) and evolved from the original diploid wheat, called einkorn (*Triticum monococcum*), through thousands of years of selective breeding and the development of tetraploid varieties [31] (Figure 2c). It has been posited that the genetic evolution, introducing new sequences into the wheat genome, could have potentially led to an increase in toxic and immunogenic epitopes responsible for the increased prevalence of CD [32] and, in general, of gluten-related disorders. A high-quality genome sequence was established from the reference wheat Chinese Spring, which made a complete set of gluten protein genes available from a single hexaploid cultivar [33,34]. Nevertheless, the large number of different wheat cultivars around the world, the high allelic variation in gluten genotypes among cultivars and the large number of immunogenic gluten epitopes make it difficult to draw firm conclusions, and the real contribution of modern wheat breeding practices to the increased prevalence of CD is still a matter of debate. Data on the reduced immunogenicity of old wheat genotypes because of the absence of the D-genome [32] have not been confirmed by more recent studies [35,36,37,38] and their health-promoting properties emerging from recent studies appear to rely on other features rather than their low immunogenicity. The macro- and micronutrient contents of ancient grains seem to decrease the risk of cardiovascular disease and metabolic syndrome, ameliorate the glycolipid profile and reduce oxidative stress and the level of pro-inflammatory cytokines. Furthermore, their consumption has been reported to curtail the extent and severity of IBS-related symptoms [39].

In the absence of convincing evidence for a role of wheat breeding in the increasing prevalence of gluten-related diseases, change in per capita consumption of wheat flour and the usage of vital gluten as a processed food additive have been postulated [40].

### 2.2. Is There a Role for Microbiota?

In recent years, the impact of gut microbiota on the loss of gluten tolerance has received increasing attention. The intestinal microbial communities represent a complex ecosystem, which plays a central role in modulating both innate and adaptive immune responses [41,42]. They are also involved in the maintenance of mucosal barrier function, which is a crucial mediator between our body and the external environment, and prevent the entry of toxic/immunogenic molecules across the intestinal wall [43,44].

In both stools and mucosal biopsies of celiac patients, a shift toward *Bacteroides*, *Clostridium* and *Escherichia coli*, with reduction in protective *Bifidobacteria*, *Firmicutes* and *Lactobacilli*, in comparison with non-celiac controls, has been described [45,46] as being partially restored by a GFD [47,48,49]. Both genetic makeup and environmental factors contribute to shaping the composition and diversity of the intestinal microbiota. Infants with a high genetic risk of developing CD harbor a higher proportion of *Firmicutes* and *Proteobacteria* and a lower proportion of *Actinobacteria* [50], resulting in an increased prevalence of pathogenic bacteria compared to those with a low risk [51]. According to the hygiene hypothesis, the decreased infectious pressure observed in industrialized countries over the last several decades should prevent the development of a functional immune system during early childhood, leading to an imbalance between pro-inflammatory and anti-inflammatory responses. Additional main drivers of microbial gut colonization, such as mode of delivery, infant feeding practice and antibiotic use, were not confirmed as risk factors for CD [52,53,54,55]. Although most studies report major differences in the composition of microbiota between celiac patients and healthy controls, a specific microbial profile cannot be identified in CD [56]. Evidence on the causal relationship between dysbiosis and disease occurrence is highly heterogeneous and controversial due to inter-individual variability, small sample sizes and different methodologies, which all hamper the interpretation of results [57]. Finally, it is still unclear whether an altered microbiota in CD patients is the cause or the consequence of mucosal inflammation [58]. The exact mechanisms by which a dysbiotic status could contribute to CD development are also still unknown and include the processing of gluten peptides, activation of innate immune response and modulation of intestinal permeability [59,60].

### 2.3. Gluten Consumption

The phenomenon of globalization is driving a revolution in food systems (supply, marketing and distribution) as well as in dietary patterns. Major changes in food culture are closely associated with urbanization, increasing incomes, capital flow and market liberalization, and are characterized by dietary convergence, a phenomenon occurring as a result of increased reliance on a narrow base of staple foods, among which the dominant staple grain is wheat [61]. Palatability, ease of large-scale cultivation, industrial food processing and low prices have all contributed to the global spread of wheat gluten consumption. Wheat production has increased sharply since 1955, showing an impressive tenfold increase in the annual rate of yield improvement, particularly in the 1960s, and gradually afterwards. This was thanks to a technology shift commonly labeled the “green revolution” [62,63]. The green revolution resulted in the development of rust-resistant semi-dwarf, high-yield wheat. Between 1980 and 2013, the world’s annual wheat yield increased by 1.41% [64]. Currently, North America maintains the leading position in the wheat gluten market, followed by Europe. The abundance of applications in the food industry and high demand for high-fiber and meat-free foods among an increasingly health-conscious and vegan/vegetarian population are considered key factors boosting the growth of the wheat gluten industry in Western countries [65]. The global wheat protein market was estimated to be valued at USD 2.04 billion in 2017 and it was foreseen to grow at a compound annual growth rate (CAGR) of 4.8% from 2017, to reach USD 2.58 billion by 2022 [66].

In highly populated, developing countries, particularly those in the Asian region, the growing middle class, adopting Western-style diets with a higher content of wheat products, have contributed to increasing its consumption [64]. Recently, global change in consumption patterns and consumer attitudes during coronavirus lockdowns, and in particular the boom of home baking, boosted a sharp increase in wheat consumption: the Spanish Minister of Agriculture, Luis Planas, revealed that sales of flour quadrupled during the third week of lockdown; Nielsen data showed that in March 2020, the retail flour sales in France, the US and Italy increased by 140, 154 and 185 percent, respectively, compared with the same period in 2019.

Vital wheat gluten (VWG) is obtained from wheat flour by removing soluble fibers and starch fractions and recovering gliadins and glutenins [67]. VWG is widely used as an additive in bakery products and pasta dough to increase yields and improve rheological, microstructure and quality characteristics [68,69]. Due to its visco-elasticity and the range of functional properties at a lower price than competitors, such as milk and soy proteins, have contributed to spreading its use in the food industry, leading to a tripled consumption since 1977, consistent with the epidemiology of CD [40].

### 2.4. Gluten Exorphins

Exogenous peptides with opioid-like activities, which include gluten exorphins (wheat), casomorphins (milk), rubiscolins (spinach) and soymorphins (soybean) [70], display regulatory functions both for the gastrointestinal and central nervous systems. In rodent behavioral models, food-derived, opioid-like peptides affect nociception, spontaneous behavior and memory. After oral, intracerebroventricular or intraperitoneal administration, some food-derived opioids also affect intestinal motility, hormone release, appetite, mucus production and local immunity [71,72]. In rats, the opioid antagonist naloxone drastically reduces the intake of preferred foods [73,74].

Enzymatic breakdown of gliadin from wheat by intestinal pepsin, leucine aminopeptidase and elastase generates morphine-like peptides, also known as gluten exorphins [75]. In healthy volunteers, early research showed that gluten exorphins induced a significant increase in gastrointestinal transit time, reversible after administration of the opioid antagonist naloxone [75,76].

In rodents, orally administered gluten exorphin A5 suppressed the endogenous pain-inhibitory system induced by socio-psychological stress and modified spontaneous behavior and learning/memory processes during several laboratory stressors, indicating that the peptides may cross the blood–brain barrier [77]. It has been suggested that the effects of food exorphins could be amplified if they are absorbed in excess through a disrupted mucosal barrier [78].

## 3. Not Only Gluten

Although general attention has focused on gluten as the only culprit of symptoms occurring in patients on a gluten-containing diet, a variety of substances, belonging to the non-gluten components of wheat, are potentially harmful, including wheat α-amylase/trypsin inhibitors (ATIs), wheat germ agglutinins (WGAs) and fructans. Moreover, glyphosate, a non-selective herbicide extensively used in farming against weeds, could play an important role due to its interference with agricultural crops.

### 3.1. Wheat α-Amylase/Trypsin Inhibitors (ATIs)

ATIs are a family of at least 11 proteins belonging to the non-gluten protein fraction. They are classified in monomeric, dimeric and tetrameric forms and represent 2–4% of total wheat protein content [79]. ATIs are contained in the endosperm of wheat seeds, where they play the multifunctional role of a natural defense against insects and parasites, inhibiting enzymes with amylase and trypsin-like activities and the regulation of starch metabolism during seed development and germination [80,81]. Identified as major allergens in baker’s asthma, as well as stimulators of innate immunity, ATIs promoted a strong innate immune response by engaging the TLR4–MD2–CD14 complex both in human and murine macrophages, monocytes and dendritic cells (DCs) and in vivo after oral or systemic challenge in mice. Furthermore, in duodenal biopsies from celiac patients in remission, ATIs induced an increase in IL-8 mRNA expression as well as a further increase in 33mer-induced IL-8 expression [82]. In line with these results, in gluten-sensitized mice expressing HLA-DQ8, ATI ingestion was recently shown to increase the inflammatory response to dietary gluten. Conversely, in ATI-fed control mice, a TLR4-mediated intestinal barrier dysfunction without mucosal damage was observed. In both cases, ATI-degrading lactobacilli decreased the inflammatory effects, suggesting new therapeutic strategies for wheat-related disorders [83]. ATIs are present and retain bioactivity in processed or baked foods. Wheat breeding practices aimed at developing high-yield, highly pest-resistant crops has led to an increased amount of ATIs in modern hexaploid wheat varieties; modern gluten-containing staples have been found to have higher levels of TLR4-activating ATIs than most gluten-free food; in mice, oral ingestion was shown to increase intestinal inflammation by activating gut and mesenteric lymph node myeloid cells [84]. Recently, a central role for ATIs has been proposed in the pathogenesis of NCG/WS within the context of a new theory which suggests a decrease in butyrate-producing intestinal bacteria as an initial trigger of the pathogenic cascade [85].

### 3.2. Wheat Germ Agglutinin (WGA)

A role of WGA as potentially responsible for many of wheat’s related, and difficult to diagnose, ill effects has been postulated. WGA belongs to the lectin group, a superfamily of carbohydrate-binding proteins present in a variety of plants with a protective role against external pathogens. It is a homodimer composed of subunits and each protomeric unit consists of four structurally homologous domains with a high degree of amino acid homology. Four interlocking disulfide bonds result in a compact, stable protein highly resistant to degradation [86]. It is present in its highest concentrations in the germ tissue of wheat kernels (up to 0.5 g/kg) [87], especially in whole wheat. Through thousands of years of selective wheat breeding to obtain increasingly higher protein content, the concentration of WGA lectin in wheat has increased proportionately, offering additional pest resistance and contributing to wheat’s global dominance as one of the world’s favored monocultures. WGA may adversely affect gastrointestinal function in various ways: it binds specifically to carbohydrates expressed by human enterocytes and immune cells and to the glycocalyx, the sialic acid coatings of the epithelial layer. In human basophils, WGA induced interleukin 4 (IL-4) and interleukin 13 (IL-13) release [88]. In an experimental model of human intestinal immune/epithelial cell interaction, it exhibited toxic and inflammatory effects by disrupting epithelial integrity and inducing the synthesis of pro-inflammatory cytokines, including interleukin 1, interleukin 6 and interleukin 8 by peripheral blood mononuclear cells (PBMCs) [89]. In murine spleen cells, WGA induced a T and B cell-independent production of interleukin 12 (IL12) and, in turn, the production of interferon gamma (IFN gamma) by T/natural killer lymphocytes [90]. In WGA-treated murine peritoneal macrophages, the production of pro-inflammatory cytokines anti-TNF alfa, interleukin 1 beta (IL-1 beta), IL-12 and IFN gamma was reported [91]. Human data on the in vivo immune-stimulatory activity of WGA are lacking [92], both in healthy subjects [93] and CD patients. However, the presence of IgG and IgA antibodies to WGA has been described, not cross reacting with gluten antigens, at higher levels in CD in comparison with patients with other intestinal diseases and healthy subjects. For this reason, a correlation with the pathogenesis of CD has been suggested [94]. Nevertheless, antibodies to wheat albumin and globulin [95], as well as to other dietary antigens such as casein, beta-lactoglobulin and ovalbumin, have also been reported in celiac patients [96] and the role of WGA in the pathogenesis of CD remains elusive.

In rodents, WGA displayed anti–nutrient effects reducing digestibility and utilization of dietary proteins: it mimicked the effects of epidermal growth factor (EGF), inducing cellular hyperplastic and hypertrophic growth [97]. In rats, it also caused damage to the intestinal brush border membrane, reduction in surface area, acceleration of cell losses, shortening of villi via binding to the villous surface [98] and cytoskeleton degradation, contributing to cell death and increased turnover.

### 3.3. Fructans

Wheat contains fructans, naturally occurring polymers of fructose molecules belonging to the fermentable oligosaccharides (fructo-oligosaccharides or fructans (FOSs), galacto-oligosaccharides (GOSs; stachyose, raffinose)), disaccharides (lactose), monosaccharides (fructose) and polyols (sorbitol, mannitol, xylitol and maltytol) (FODMAP) group [99]. FODMAP was named in 2005 by the Monash group in a paper on the link between a FODMAP-rich diet and lifestyle in Crohn’s disease patients [100]. FODMAPs can be found in a wide range of staple foods such as fruits, vegetables, legumes and cereals, honey, milk and dairy products and sweeteners [100,101].

Owing to their small size, they are osmotically active and rapidly fermented by gut bacteria in the large intestine [102]. The combination of osmotic activity with fluid retention within the intestinal lumen and gas production by fermentation of oligosaccharides and polyols induces a variety of symptoms. These include bloating, abdominal pain and diarrhea [103].

Fructans increase the tolerance of wheat to drought and cold [104]. Their content in wheat is highly variable and depends on the final product; no significant difference was found between wheat breads and the gluten-free counterparts (approximately 1% in both) [105,106]. Furthermore, gluten-free products, like corn, can have quite a large amount of FODMAPs, mainly fructans, galactans and fructose [105].

### 3.4. Wheat Glyphosate

Glyphosate is a non-selective herbicide and, since the late 1970s, one of the most extensively used in farming against weeds that interfere with agricultural crops like soy, corn and wheat [107]. A role as a causal factor for the worldwide increase of CD incidence was initially proposed, based on the glyphosate effects on intestinal microbiota, micronutrient absorption, enzymatic detoxification and serotonin signaling, as well as on the increased risk of non-Hodgkin’s lymphoma in celiac patients [108]. Nevertheless, the paper received criticism for being merely speculative. Other studies based on in vivo and in vitro animal models [109,110] and on cultured human and rat intestinal cell lines [111] have postulated a negative impact of glyphosate on intestinal microbiota, barrier properties and motility.

A strong limitation of these studies is that most of them, due to obvious ethical reasons, have been conducted on experimental models. In the absence of robust evidence, the causative link between glyphosate and gluten-related intestinal disorders remains hypothetical.

## 4. Gluten-Related Disorders

Wheat proteins are recognized as environmental triggers of two well-established immune-mediated disorders, CD and wheat allergy (WA). Furthermore, a gluten-related condition much debated in these last years is non-celiac gluten sensitivity (NCGS).

### 4.1. Celiac Disease (CD)

CD is a chronic small bowel enteropathy occurring in genetically susceptible individuals where dietary gluten peptides elicit both innate and adaptive Th1-driven immune responses, amplified by transglutaminase-mediated synthesis of negatively charged glutamate residues from glutamine [26]. Access of immunogenic gluten peptides to the small intestine lamina propria is fostered by gluten-induced up-regulation of zonulin, a modulator of intestinal tight junctions [112]. Besides the CD-predisposing HLA DQ2/DQ8 haplotypes, genome-wide association studies have identified 39 non-HLA loci affecting CD [113]. Environmental factors may also be of importance for CD development. A correlation with some viral infections, especially during early childhood, has been suggested, including rotavirus, reovirus, enterovirus A and B and acute respiratory infections [114,115,116]. Based on the evidence that the microbiota affects the immune response [117] and indications of microbiota alterations in celiac patients, a correlation between dysbiosis and the risk of developing CD has been postulated [118,119,120]. The role of other environmental factors, such as infant feeding practices, mode of delivery, age of gluten introduction and amount of gluten in early life or exposure to antibiotics, has not been confirmed or has given contradictory results [54,121,122,123,124,125,126].

CD is diagnosed more frequently in females with a F:M ratio of 2:1 on average and an onset following a bimodal age distribution, with an initial peak in the first 2 years of life and a second peak in the second or third decade, although about 25% of all diagnoses occur at the age of 60 years or more [127]. Clinical presentation ranges from virtually asymptomatic cases, despite typical mucosal damage (silent CD), to severe malabsorption and includes a variety of mild to severe intestinal and/or extraintestinal symptoms [128], especially involving iron and bone metabolism, central and peripheral nervous systems and reproductive system. Multiple autoimmune diseases have been described in association with CD, most commonly autoimmune thyroiditis, type 1 diabetes and liver and rheumatologic disorders [129]. Diagnosis of CD relies on the assessment of specific circulating antibodies and on the demonstration of duodenal mucosal damage (ranging from lymphocytic enteritis to severe villous atrophy); in selected cases, HLA DQ typing is recommended. According to the European guidelines, duodenal biopsies can be avoided in symptomatic children with high titer serology [130]. Owing to a misleading clinical presentation and/or lack of clear-cut diagnostic tests in many patients, diagnosis of CD requires time and expertise to properly combine clinical, serologic, histologic and genetic data. About 1% of patients, especially those with late diagnosis, low adherence to diet and HLA DQ2 homozygosis, develop pre-malignant or malignant complications (refractory CD, ulcerative jejunoileitis, enteropathy-associated T cell lymphoma (EATL), small bowel adenocarcinoma) [131,132] or hyposplenism [133].

### 4.2. Wheat Allergy (WA)

**Figure 3:**
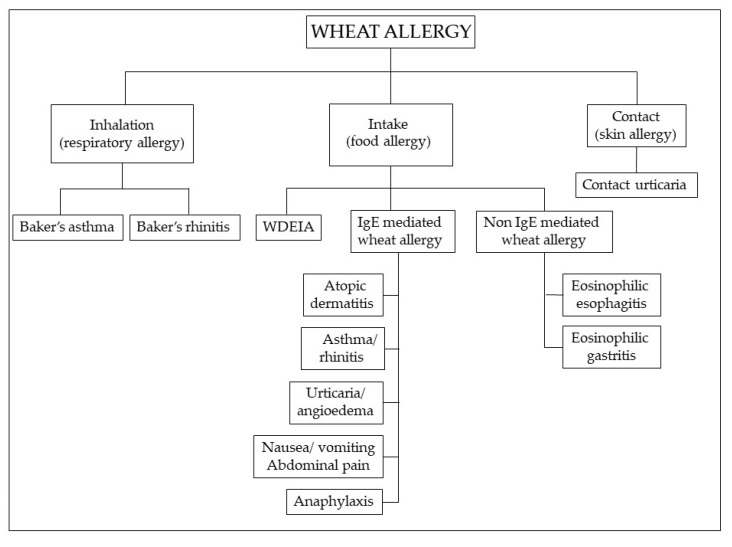
Classification of wheat allergy depending on the route of exposure and the underlying immunologic mechanism. Abbreviations: WDEIA: wheat-dependent, exercise-induced anaphylaxis.

WA has a prevalence in the range of 0.2% to 1% [134]. Although more common in children [135], most of whom outgrow it by the age of 16 years [136], symptoms may occur at any stage of life, including later adulthood. The prevalent immune mechanism is IgE mediated, but non-IgE-mediated reactions are also described [137,138], characterized by chronic infiltration of eosinophils and lymphocytes in the gastrointestinal mucosa (Figure 3).

WA is a classic food allergy characterized by cutaneous, gastrointestinal or respiratory manifestations. These include wheat-dependent, exercise-induced anaphylaxis (WDEIA), which results from the combination of wheat ingestion and physical exercise, baker’s asthma and rhinitis, occurring after inhalation of wheat and cereal flours, which is one of the most common occupational allergies and contact urticaria [139]. Children with WA mainly display moderate-to-severe atopic dermatitis; wheat ingestion may also elicit IgE-mediated urticaria, angioedema, bronchial obstruction, nausea and abdominal pain, or even severe systemic anaphylaxis [140]. In adults, the most common variant is WDEIA, where symptoms range from urticaria to systemic reactions, including anaphylaxis [141]. Multiple allergens are involved in WA [142]: sera from patients with baker’s asthma and rhinitis react with amylase inhibitors, germ agglutinin, peroxidase and non-specific lipid transfer proteins (LTPs) [143]; WDEIA is induced by ω5-gliadins [144]; IgE from patients with atopic dermatitis, urticaria and anaphylaxis are reactive with α, β, γ, ω-gliadins, and low and high molecular weight subunits. Over 50% of patients with urticaria have IgE to ω5-gliadin [145]. The first-level diagnostic tests for WA are in vitro specific immunoglobulin E (sIgE) assays and skin prick tests (SPTs), which, however, have a low predictive value. Functional tests (bronchial challenge test in baker’s asthma and food challenge in food allergy) are considered the diagnostic gold standard for WA [146], but they are impractical and potentially dangerous. Molecular-based allergy (MA) diagnostics and a flow cytometry-assisted basophil activation test (BAT), an in vitro functional test for the diagnosis of immediate type allergy for patients at risk of severe anaphylactic reactions, are a novel diagnostic approach to allergic disorders that in some cases may represent an effective alternative to the in vivo functional tests [141,146].

### 4.3. Non-Celiac Gluten/Wheat Sensitivity (NCG/WS)

For the sake of simplicity, the wide range of intestinal and extra-intestinal symptoms occurring after the ingestion of gluten-containing food in subjects who do not have either CD or WA has been collectively defined as NCGS, more recently renamed non-celiac wheat sensitivity (NCWS) [147,148]. The occurrence of gluten-related disturbances beyond CD was initially reported in 1980 [149] and later in 2000 [150], but it was only in 2011 that NCG/WS took center stage as part of the spectrum of gluten-related disorders [151]. Since then, rising public interest and a growing body of research have fueled constant debate regarding this issue, with an overwhelming discrepancy between media messages and scientific citations [17]. The internet, the popular press, marketing claims and celebrities endorsing their gluten-free choices represent common sources of information with no reliable scientific evidence. The clinical picture is heterogeneous and non-specific, ranging from “IBS-like” symptoms (diarrhea, constipation, bloating, nausea and epigastric pain) to extra-intestinal manifestations (malaise, anxiety, fibromyalgia, skin rash, tiredness and chronic fatigue, “foggy mind” and headache) [152]. Owing to the lack of specific biomarkers, prevalence data in the general population are highly variable, ranging between 0.6% and 10.6% [92]. Recently, in NCGS, a significant increase in anti-gliadin IgG2 antibodies was described in comparison with healthy controls and an increase in anti-gliadin IgG4 antibodies was reported in comparison with CD and healthy controls, suggesting their potential role as diagnostic biomarkers [153]. Furthermore, evidence was provided for an overexpression of selected miRNA in the intestinal mucosa and peripheral blood leukocytes (PBLs) of NCWS patients if compared to symptomatic controls with functional dyspepsia or CD. Hsa-miR-30e-5p proved to be the best predictor of NCWS vs. CD in biopsies and vs. controls in PBLs [154]. The absence of validated diagnostic criteria also explains the high rate of self-diagnosis [155,156] as well as the impact of patients’ perception of symptoms and of the nocebo effect on the interpretation of study results [157]. In an attempt to establish the actual role of gluten, double-blind placebo-controlled (DBPC) gluten challenge trials have been suggested. Molina-Infante and Carroccio, in order to evaluate the accuracy of this approach, analyzed 10 of these trials including 1312 adults. The studies were different regarding the duration of the gluten challenge (1 day–6 weeks) and the wash out period (3 days–2 weeks), gluten daily dose (2–52 g) and the kind of placebo administered (gluten-free products, xylose, whey protein, rice or corn starch containing fermentable carbohydrates). Most of the trials reported that gluten was able to significantly aggravate symptoms when compared to placebo, but only 38 out of 231 patients (16%) specifically reacted to gluten. Moreover, a nocebo effect (similar or increased symptoms after placebo administration) was observed in 40% of the patients [158]. The heterogeneity of these studies should also be highlighted because this potentially affected the results, and these data prompt some doubts as to the role of gluten as a “trigger” food of symptoms because more than 80% of NCGS diagnosed on the basis of a positive response to a GFD cannot be formally diagnosed after a DBPC trial (not performed in all the studies according to the protocol recommended by the Salerno Experts [147]) and this sheds light on the possible importance of the so-called “nocebo” effect which cannot be excluded in studies involving a dietary approach.

Apart from gluten, potential dietary triggers include non-gluten wheat components such as ATIs, WGA and fructans. ATIs are highly resistant to intestinal proteolitic degradation and have been identified as strong activators of innate immune responses in human and murine macrophages, monocytes and DCs, eliciting the release of proinflammatory cytokines via the activation of TLR4 [82]. In mice, ATIs showed an additive effect on pre-existing low-level intestinal inflammation, with stimulatory activity increasing from the proximal intestine to the ileum and colon [84]. Involvement of an adaptive immune response by the migration of DCs to mesenteric lymph nodes and interaction with primed T cells could exacerbate the ongoing inflammation [84]. In vitro studies and in vivo animal models showed that WGA induces the release of pro-inflammatory cytokines and epithelial barrier disruption [89]. In vivo human studies are needed to better support the role of ATIs and WGA as triggering factors of NCG/WS. Unlike ATIs and WGA, fructans induce a variety of IBS-like symptoms, including bloating, abdominal pain and diarrhea, due to a combination of osmotic activity with fluid retention within the intestinal lumen and gas production by fermentation [102,159]. In a DBPC study, Biesiekierski et al. reported that patients with NCGS did not exhibit statistically significant effects after gluten was added to the diet in the presence of a low content of FODMAPs, indicating that symptoms may be due to fructans rather than gluten [160]. Another DBPC crossover study in patients with self-reported NCG/WS showed that fructans (rather than gluten) were more likely to induce symptoms, with no effect of gluten challenge [161]. However, Volta et al. argued that the authors had enrolled self-diagnosed NCG/WS, some extra-GI symptoms typical of NCG/WS had not been included in the evaluation, anti-gliadin IgG antibodies had not be assessed and only the prevalence of Hashimoto’s thyroiditis had been reported as an autoimmunity marker [162,163]. Hence, numerous patients with NCG/WS could have a diagnosis of IBS.

Exposure to hidden ingredients such as chemical additives and preservatives, commonly added to processed food as antimicrobial agents or to improve appearance, flavor or texture, might contribute to generating intestinal symptoms by inducing pro-inflammatory cytokines, altering gut microbiota composition and disrupting the mucosal barrier [164].

In the absence of a definite mechanism of action, the pathogenesis of NCG/WS remains a matter of debate; IgE-independent WA involving mast cells, eosinophils and other immune cells [165] has been postulated on the basis of past or current history of food allergy [166], eosinophil infiltration of the intestinal mucosa and in vitro basophil activation induced by food antigens in patients with NCG/WS diagnosed by DBPC challenge [167]. Furthermore, an increase in mucosal lymphocytes has been detected in some NCG/WS patients [167,168]. In particular, infiltration with innate lymphocyte-1 cells producing IFN-γ and responsive to a wheat-free diet has been described in the rectal mucosa of patients [169]. Current evidence suggests a complex interplay among systemic immune response, impaired intestinal barrier function and dysbiosis [170]. The early findings of a reduced intestinal permeability [168] in NCG/WS patients have not been confirmed: further studies have definitely shown intestinal epithelial damage leading to compromised barrier function [171,172] and microbial translocation from the lumen to the intestinal mucosa, resulting in a systemic, mainly innate, immune response [173,174]. In wheat-sensitive patients, altered expression of markers of an innate immune response has been described [168,175]. Positive serology for native gliadin in a proportion of patients [176] suggested a concomitant role of an adaptive immune response [173]. In wheat-sensitive individuals, Uhde et al. demonstrated increased serum markers of systemic innate immune activation as well as B cell response to microbial antigens associated with markers of intestinal epithelial cell damage as indicators of the translocation of microbial products across the intestinal mucosa, reversible on a GFD [177]. Based on the recognized involvement of an impaired intestinal permeability in the pathogenesis of NCG/WS [112,178] and on the studies regarding the immune-stimulating activity of ATIs [82,84], a new hypothesis has been formulated implicating the Western diet and lifestyle which, inducing dysbiosis with low levels of intestinal butyrate-producing bacteria, could lead to a vicious circle involving a disrupted intestinal barrier function, microbial lipopolysaccharide (LPS), decreased intestinal alkaline phosphatase (IAP) and intact ATI translocation [85].

In recent years, the overlap between NCG/WS and IBS has drawn increasing attention [92,179]; an Italian multicenter study found IBS in about 50% of these patients [152]. With an estimated 11.2% worldwide prevalence [180], IBS is the most prevalent functional gastrointestinal disorder (FGID) [181], causing a significant impairment of patients’ quality of life and productivity with a high social and economic impact [182]. Shared symptoms between IBS and NCG/WS are abdominal pain, altered bowel habits, bloating and/or extra-intestinal symptoms [183,184,185]. Food is regarded as a precipitating factor of symptoms by many IBS patients [186,187]. In the absence of specific tests, the diagnosis of IBS [188] essentially relies on symptom assessment, standardized in the Rome IV Criteria [189]. In recent years, a low-FODMAP diet (LFD) involving a global restriction of FODMAP intake followed by gradual re-introduction, according to individual tolerance and under the supervision of an expert dietician [190], has been widely employed for IBS treatment [191] and it is currently regarded as effective in reducing IBS symptoms, according to several studies [192,193,194,195].

Unfortunately, only a few studies dealing with LFDs have been based on randomized placebo-controlled double-blind trials [190]. A recent systematic review and meta-analysis of randomized controlled trials (RCTs) examining the efficacy of an LFD and GFD in IBS provided evidence that an LFD is more effective than a GFD in reducing IBS symptoms [194], although the evidence is of very low quality. The authors justified the very low quality of evidence regarding LFD efficacy because of the heterogeneity of the studies, i.e., the different types of comparators used in the different studies and the low number of patients reporting global symptom improvement (189 out of 397 patients, whereas the GRADE system would require at least 300 patients) [196]. The authors also underlined that the problems could be solved if further trials were be carried out using similar comparator groups in order to provide more data [194]. Unfortunately, there is a problem of economic resources because it is quite difficult to find subjects interested in financing such studies [190].

**Figure 4:**
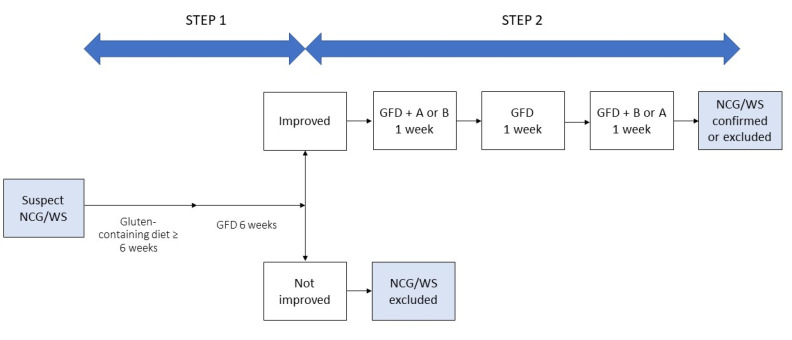
Diagnosis of non-celiac gluten/wheat sensitivity (NCG/WS), modified from Catassi, 2015 [147]. GFD: gluten-free diet.

Differentiating IBS from NCG/WS can be cumbersome and needs to take into account the overlapping clinical picture, the lack of specific biomarkers, the putative role of the same dietary triggers and the influence of patients’ perceptions. Waiting for validated biomarkers able to obtain a differential diagnosis, some authors suggest that the patients’ opinions on the role of gluten in precipitating their digestive symptoms could be used as criteria to distinguish NCG/WS from IBS, although the gluten-related nocebo effect and the questionable reliability of patients in identifying the dietary culprit of their symptoms should be taken into account. With the aim of overcoming the limitation of a diagnosis merely based on exclusion criteria and to standardize the diagnostic procedure, an international group of experts elaborated the Salerno Experts’ criteria for the diagnosis of NCG/WS based on a double-step approach. In Step 1, after exclusion of CD and WA, patients start a six-week gluten-containing diet and report their symptoms according to a modified version of the Gastrointestinal Symptom Rating Scale (GSRS). Then they start a GFD for at least six weeks. A decrease of at least 30% of the baseline score is considered a positive response. Step 2 includes a one-week challenge (GFD and gluten or placebo) followed by a one-week washout of strict GFD and a crossover to the second one-week challenge. A variation of symptoms of at least 30% between gluten and placebo challenge discriminate a positive from a negative result [147] (Figure 4).

Although a DBPC approach represents the gold standard for a rigorous identification of NCG/WS [147], it is cumbersome and impractical for clinicians. Furthermore, wheat is commonly identified as a trigger when IBS patients are specifically interviewed [197,198,199]. The clinical effects of the aforementioned compounds could explain the overlapping symptoms of NCG/WS and IBS, as well as the causative role of wheat in a subgroup of IBS patients, and their symptomatic improvement after wheat elimination [92,183,200]. In this regard, the term “wheat-sensitive IBS” has been coined to describe patients who meet the Rome IV criteria for IBS and report gluten/wheat-related symptoms [92]. In everyday practice, it is not easy to clearly distinguish NCG/WS from “wheat-sensitive IBS”, which represents a “gray zone” where different concepts and symptoms can overlap. As a consequence, choosing the most appropriate dietary intervention within this overlap is challenging. However, the usefulness of such a distinction from a clinical point of view could be relatively unimportant. The GFD can be considered in patients with self-reported gluten/wheat-dependent symptoms, especially if associated with extra-intestinal manifestations, most likely not induced by fructans [6,92,201]. In non-responders or partial responders to a GFD, an LFD could be considered as a second-line treatment. Moreover, we think that in patients not reporting gluten/wheat as trigger of their symptoms and referring symptoms more related to FODMAPs other than fructans, an LFD could be the first dietary option. In any case, irrespective of the type of dietary approach, the patients’ preferences must be taken into account.

## 5. Conclusions

In the last 30 years, the GFD and related GF products have gained increasing popularity. These have been supported by marketing campaigns, athletes and celebrities, media messages and social networks. Nevertheless, real knowledge of gluten and GF-related implications for health is scarce in the population.

The role of potential causative factors in the increasing prevalence of CD is under debate. Modern wheat breeding practices have not been confirmed; per capita vital gluten consumption, variation in the amount and types of wheat intake and agronomic practices affecting wheat protein content have been proposed as contributors to the toxicity of wheat in genetically susceptible individuals [40]. Although general attention has focused on gluten as the only culprit of symptom occurrence in non-celiac patients on a gluten-containing diet, the role of a variety of compounds (ATIs, WGA, fructans and glyphosate) belonging to the non-gluten components of wheat appears to be prevalent.

Despite the wide acceptance of the term by the scientific community, the existence of NCG/WS as distinct entity has been questioned, and more properly could be regarded as a collective term for a variety of different conditions where gluten is directly involved only in a small minority of patients [158]. Likewise, the pathogenesis seems to be multifactorial, including innate immune response, altered mucosal barrier function and dysbiosis [202]. In the absence of specific diagnostic markers, and under the influence of marketing and media claims, a high rate of self-diagnosis occurs [152,156].

A GFD might be an appropriate dietary approach for patients with self-reported gluten/wheat-dependent symptoms. An LFD should be the first dietary option for patients referring symptoms more related to FODMAPs than gluten/wheat, and the second-line treatment for those with self-reported gluten/wheat-related symptoms who do not respond to a GFD. In any cases, a personalized approach, regular follow-up and the intervention of an expert dietician are recommended.
